# Clinical study of vacuum phenomenon in closed pelvic fracture

**DOI:** 10.1186/s13018-023-03674-z

**Published:** 2023-03-25

**Authors:** Rui-Feng Yang, Shu-Ming Huang, Quan-Zhou Wu, Fang Ye, Shu-Hua Lan

**Affiliations:** Department of Orthopedics, Lishui Municipal Central Hospital, Lishui, 323000 Zhejiang People’s Republic of China

**Keywords:** Vacuum phenomenon, Vacuum sign, Air, Pelvic fracture, Fragility fractures of the pelvis

## Abstract

**Background:**

This study aimed to examine the prevalence and clinical findings of the vacuum phenomenon (VP) in closed pelvic fractures.

**Methods:**

We retrospectively reviewed 352 patients with closed pelvic fractures who presented to our institution from January 2017 to December 2020. Pelvic fractures were diagnosed by plain radiography and computed tomography (CT). The default “bone window” was used for inspection in the cross section. Electronic medical records were consulted by two orthopedic physicians to obtain patient information. The VP of pelvic fracture, fracture classification, injury mechanism, and image data were evaluated, and the demographic parameter data were statistically analyzed. The follow-up time was 12–18 months.

**Results:**

Among them, 169 were males and 183 were females with ages ranging from 3 to 100 years, with an average of 49.6 ± 19.3 years. VP in pelvic fractures was detected by CT in 109 (31%) of the 352 patients with pelvic fractures. Patients were divided into the high-energy trauma group (278 cases) and fragility fractures of the pelvis (FFP) group (74 cases) according to the injury mechanism. In the high-energy trauma group, 227 cases were treated surgically and 201 cases had bony healing. The healing time was 9.8 ± 5.3 weeks. In the FFP group, 54 cases were treated surgically and 49 cases had bone healing. The healing time was 9.3 ± 3.8 weeks. Fractures progressed in nine patients. VP was mostly located in the sacroiliac joint in our study.

**Conclusions:**

The incidence of VP in pelvic fractures is statistically high and is affected by many factors, such as examination technique, joint position, population composition, etc. Therefore, the VP is not a reliable sign of pelvic injury. Clinically, we need to determine the nature of VP in conjunction with gas patterns, laboratory tests, history, and physical examination.

## Introduction

At present, the vacuum phenomenon (VP) is often used to describe the gas-like density region. VP (acute VP) may be caused by the rapid increase in joint space volume, or it may be the long-term gas accumulation (subacute or chronic VP) [1]. VP formation can be explained by gas solubility, pressure–volume relationship, and human physiology [1]. Cavitation is called a VP in radiology in he human body [2]. The decrease in solubility will cause the gas to separate from the solution. The combination of low nitrogen solubility and low nitrogen metabolism in the human body is the main reason for VP formation [1]. VP has been detected and confirmed by ultrasound, plain film, computed tomography (CT), and magnetic resonance imaging [3–9].

Clinically, the pathology related to VP mainly includes normal joint movement, intervertebral disk or joint degeneration, and trauma [2]. Most of them are related to chronic degenerative changes [10]. The presence of VP in degenerative disks or joints is considered the result of motor effect accumulation (effects on tissues). This means that VP is the result of repeated minor joint traumas. VP is most often associated with degenerative arthropathy, but it is also associated with other pathologies. They include degenerative joint disease, fractures, metastases, abscesses, osteomyelitis, multiple myeloma, surgical air introduction, joint effusion, Schmorl’s nodules, and ligament tears [1, 2]. Additionally, VP is found in Panner’s disease [11] and hip impact signs [12].

Spinal VP, including intervertebral disk, has been reported in many pieces of literature, but the literature rarely reported VP related to closed pelvic fracture. A scholar once reported a VP case related to pubic nonunion [13]. The composition of the gas includes up to 90% of nitrogen, oxygen, carbon dioxide, and a small number of trace gases. It was initially considered to be related to avascular necrosis of bone, and the root cause remains under debate[1]. This study aimed to investigate the prevalence and clinical significance of VP in closed pelvic fractures.

## Data and methods

### clinical data

This study was approved by the ethics committee of Lishui Municipal Central Hospital. All patients signed the informed consent form and agreed to participate in the clinical trial. This study follows the ethical standards of the 1964 Declaration of Helsinki[14].

The clinical data of patients with closed pelvic fractures who were hospitalized in our hospital from January 2017 to December 2020 were retrospectively analyzed. During the study, patients with pelvic fractures were evaluated by Dr plain film and CT according to their medical history and physical examination. Exclusion criteria included patients who did not receive pelvic CT scan upon initial evaluation and patients with acetabular fracture, urinary system injury or colorectal injury, infection, periprosthetic fractures around the pelvis, and malignant tumors. Finally, this study included 352 cases.

The investigation team includes two senior orthopedic attending physicians and a senior radiologist certified (authorized) by the committee. The image data are independently analyzed and diagnosed by an orthopedic physician and a radiologist. Any inconsistency will be determined after discussion. The decision shall be made by another orthopedic surgeon with higher qualifications if disagreement remains. The treatment strategy was decided by the senior attending physician.

Two orthopedics physicians obtained demographic data by consulting electronic medical records and evaluated demographic data, including age, gender, injury mechanism, fracture type, history of osteoporosis detected by dual-emission x-ray absorptiometry, gas accumulation location detected by CT, surgical history of pelvic fracture, and bone healing.

Sacral fractures were classified according to the Denis classification [15]. Pubic fractures were classified according to the Nakatani classification [16]. Fragility fractures of the pelvis (FFP) are caused by non-invasive or low-energy trauma, including falling from standing height, and are classified according to the Rommens Hofmann classification. Fracture progression (FP) was defined as the progression of one or more grades of fractures according to FFP classification, or from unilateral to bilateral pubic branch or sacral fractures, or previous FFP [8]. Pelvic fractures caused by high-energy trauma were classified according to the Orthopedic Trauma Association (AO/OTA) classification [17].

Fracture healing was determined according to the Chinese Medical Association fracture healing criteria [18] as (1) no local pressure pain, no longitudinal percussion pain, and no abnormal activity; (2) x-ray revealed blurred fracture line, continuous bone scab through the fracture line was judged; (3) functional measurement: the upper limb could lift 1 kg of weight flat for 1 min and the lower limb could walk continuously for 3 min with external fixation removed; (4) no complications such as incision infection, joint function change, vascular nerve injury, etc. Bone union was defined by pain-free gait or sclerosis on radiographs.

### Imaging data

A 2-mm thick spiral CT scan was obtained using a 64- or 256-slice multi-slice spiral CT scanner depending on the patient’s visit time and reviewed on the digital image archiving and communication system (PACS) using a standard resolution monitor. The default “bone window” is used in the CT cross section for examination. The positive result of VP was defined as the gas-forming area with a minimum area of 1 mm [2, 19] and a CT value of at least − 300 Hounsfield units. The presence of VP in the fracture site and surrounding soft tissue was coded.

### Statistical analysis

Statistical Package for the Social Sciences version 24.0 (SPSS Company, USA) was used to statistically analyze the measurement results. The continuous variables with normal distribution were expressed as mean ± standard deviation (SD) and analyzed by paired *t*-test, Wilcoxon rank sum test, and Mann–Whitney test. The chi-square or Fisher exact test was used to analyze categorical variables, as appropriate. *P*-values of < 0.05 were considered a statistically significant difference.

## Results

### Clinical data of included patients

All patients were followed up for 12–18 months by outpatient or telephone. Among 352 patients with pelvic fracture, 169 were male and 183 were female, aged from 3 to 100 years, with an average of 49.6 ± 19.3 years. The included patients were divided into the VP and control groups according to the presence or absence of VP. A total of 109 (31%) cases had VP detected by CT. The average age of patients in the VP and control groups was 52.7 ± 16.1 and 48.7 ± 20.1 years, respectively. No significant difference was found between the two groups in gender, age, injury mechanism, and hospitalization days (*P* > 0.05) (Table 1).

**Table 1:** Clinical data of included patients

	VP group (*n* = 109)	Control group (*n* = 243)	*P*
*Gender*			
Male	40	129	0.743
Female	69	114	
*Age (years)*	52.7 ± 16.1	48.7 ± 20.1	0.340
< 40	25	79	
40 ~ 60	34	93	
> 60	50	71	
*Injury mechanism*			
Accident	48	103	0.490
Tumble	23	43	
Falling accidents	21	52	
Heavy object crush	5	13	
Sports injury	3	9	
Bicycle fall injury	2	6	
Others	7	17	
*Smoking history*			0.004
Yes	23	52	
No	86	191	
*Hypertension*			0.214
Yes	45	82	
No	64	161	
*Diabetes*			0.266
Yes	28	48	
No	81	195	
*History of alcohol abuse*			0.694
Yes	18	46	
No	91	197	
*Combined with Other fractures*			0.937
Yes	16	36	
No	93	207	
*Osteoporosis*			0.293
Yes	33	59	
No	76	184	
*Body mass index (kg/m*^*2*^*)*			0.996
< 18	5	11	
18.5–23.9	92	206	
≥ 24	12	26	
*Length of stay (day)*	13.9 ± 8.8	12.5 ± 9.0	0.450
*Survival rate (%)*	100	100	NS

Data are mean ± SD or N. VP, vacuum phenomenon

### Comparison between the high-energy trauma group and the FFP group

Patients were divided into the high-energy trauma group (278 cases) and the FFP group (74 cases) according to the injury mechanism. According to AO/OTA classification, 58 cases of type a fracture, 183 cases of type B fracture, and 37 cases of type C fracture were classified in the high-energy trauma group. Among them, 227 cases were treated surgically and 201 cases had bony healing. The healing time was 9.8 ± 5.3 weeks.

According to the FFP classification of fragile pelvic fractures, 23, 38, 10, and 3 cases were classified as type I, II, III, and IV fractures, respectively. Nine patients experienced FP. Among them, 54 cases were treated surgically and 49 cases had bone healing. The healing time was 9.3 ± 3.8 weeks.

Additionally, no significant difference was found in bone healing time between patients in the high-energy trauma and FFP groups (9.8 ± 5.3 weeks vs. 9.3 ± 3.8 weeks) (*P* > 0.05) (Table 2).

**Table 2:** Comparison between the high-energy trauma group and the FFP group

	high-energy trauma group (*n* = 278)	FFP group (*n* = 74)	*P* value
AO/OTA classification,			
A	58		
B	183		
C	37		
FFP classification			
I		23	
II		38	
III		10	
IV		3	
Operative treatment	227	54	
Fracture healing	201	49	
Healing time (weeks)	9.8 ± 5.3	9.3 ± 3.8	< 0.05

Data are mean ± SD or N. FFP, fragility fractures of the pelvis

## Discussion

This study examined the VP phenomenon in 352 patients with pelvic fractures and revealed a 31% VP phenomenon incidence in pelvic fractures that mainly occurred in the sacroiliac joint location. Our study will help provide data to support the clinical study of the VP phenomenon in pelvic fractures.

VP in sacroiliac joint with sacrum fracture and VP in sacrum fracture support the diagnosis of dysfunction fracture [20]. VP in sacroiliac insufficiency fractures is related to the connection of adjacent air that contains sacroiliac joints. However, subsequent studies revealed no direct relationship between VP and sacroiliac joint trauma or sacroiliac insufficiency fracture [21, 22]. At present, the vacuum around the sacroiliac joint as the potential evidence for the early stage of sacroiliac insufficiency fracture is controversial. This study aimed to investigate the prevalence and clinical significance of VP in closed pelvic fractures.

The average age of our population is 49.6 ± 19.3 years old, which is consistent with the 51.5 years old and 50.6 ± 18.5 years old reported by Faflia and Lo, respectively [23, 24]. This study revealed no significant difference between the average age of patients with (52.7 ± 16.1 years) and without VP (48.7 ± 20.1 years). The detection rate of traumatic female pelvic VP in our study was 37.7% (69/183). This study revealed a higher incidence of VP in females than in males but the difference was insignificant. Huang et al. reported that the occurrence rate of sacroiliac joint vacuum sign was 11.18%, including 5.61% in males and 19.84% in females [25]. Ying et al. studied 718 sacroiliac joints of 359 patients, and 343 of them had vacuum signs, with an occurrence rate of 47.8%, including 34.51% in males and 67.12% in females [26]. We speculate a certain correlation between the gender difference in the incidence of pelvic VP and pelvic morphology. The sacroiliac joint morphology can be divided into the “s” type and the linear type. The male is mainly linear, and the female is mainly S-shaped. The difference in pelvic morphology can affect the biomechanical environment and the VP occurrence. Further research is needed. Chronic microvascular diseases, such as atherosclerosis, are long-term coexisting diseases that increase with age and may interfere with the blood supply of joints [1]. Hypertension and diabetes are well-known risk factors for atherosclerosis. Decreased perfusion due to atherosclerosis may impair the clearance of nonmetabolic gases. However, this study revealed no significant difference in the prevalence of pelvic VP between hypertension and diabetes. The presence of chronic microvascular disease does not affect VP pathogenesis. Further research is needed[27].

At present, CT is considered the gold standard for detecting VP[1]. VP can be observed in approximately 2% of spinal x-ray films and almost 20% in elderly patients [28]. Many studies revealed no VP on x-ray plain film [4, 19]. VP is preliminarily considered to be related to intestinal gas interference and irradiation position. Conducting imaging examination only through x-ray film is far from enough for the observation of VP in pelvic fracture. Our study detected VP in 31% (109\/352) of patients with closed pelvic fractures by CT. The detection rates of males and females were 23.6% and 37.7%, respectively, which was slightly different from the prevalence rates reported by Faflia et al. [23] (males: 25.8% and females: 31.7%) and Lo et al. [24] (men 27% and women 41%). The main reason is that the sample size consisted mostly of patients with trauma. The participants in this study generally experienced high-energy impacts. A complete joint capsule is required to produce intra-articular gas. However, the high-energy impact may increase the frequency of joint capsule rupture, resulting in a decreased detection rate of VP in the articular cavity. The difference in injury mechanisms among participants may be the reason why our results are slightly different.

**Fig. 1:**
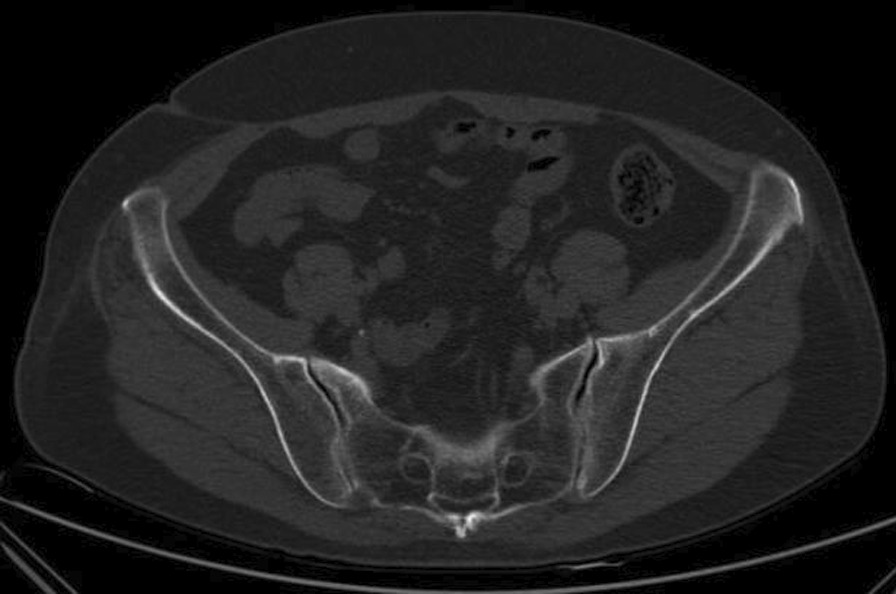
A dark line of linear gas density can be seen in bilateral sacroiliac joints of patients with trauma

**Fig. 2:**
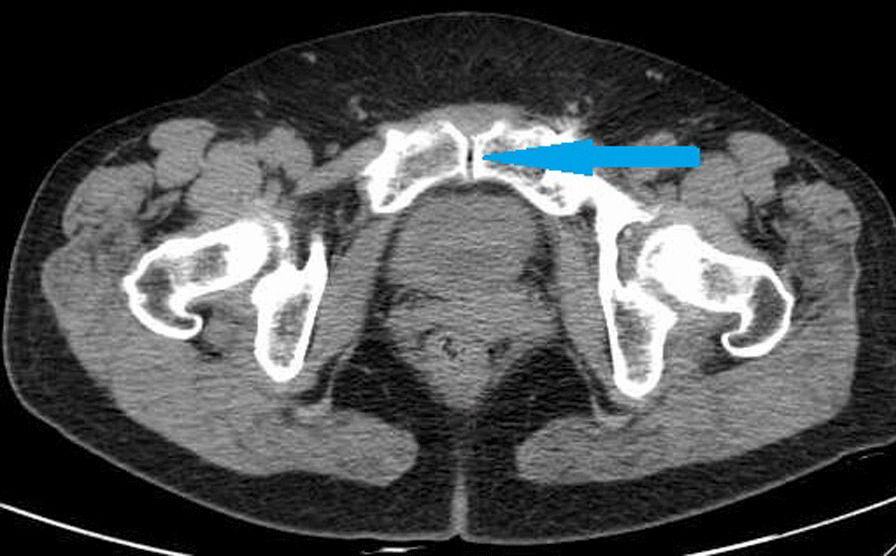
The vacuum phenomenon is located at the pubic symphysis

**Fig. 3:**
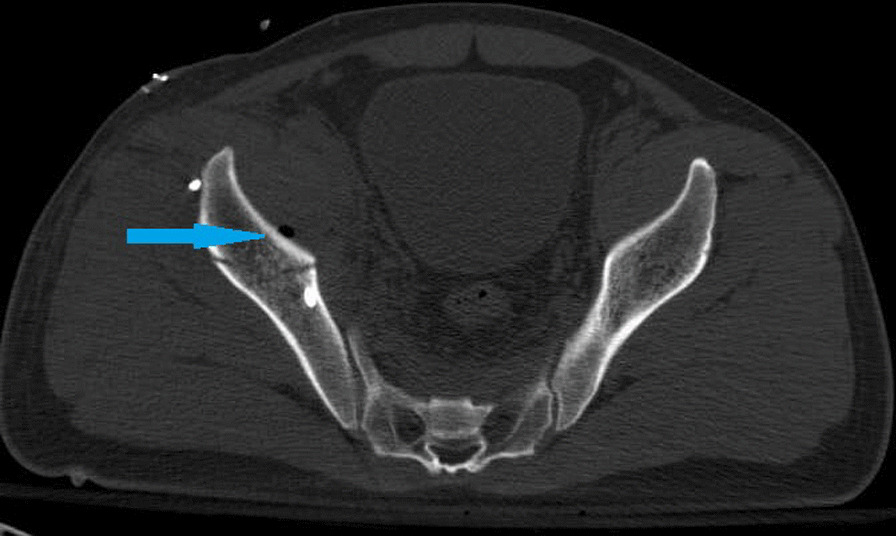
The vacuum phenomenon was located near the fracture of the right iliac bone

**Fig. 4:**
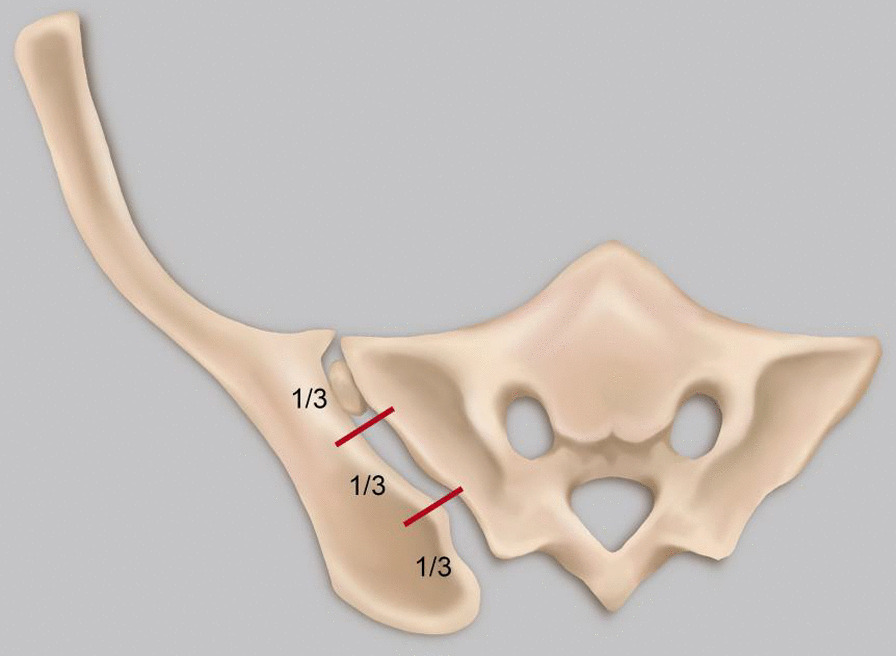
The upper, middle, and lower points of the sacroiliac joint for zoning and observation

**Fig. 5:**
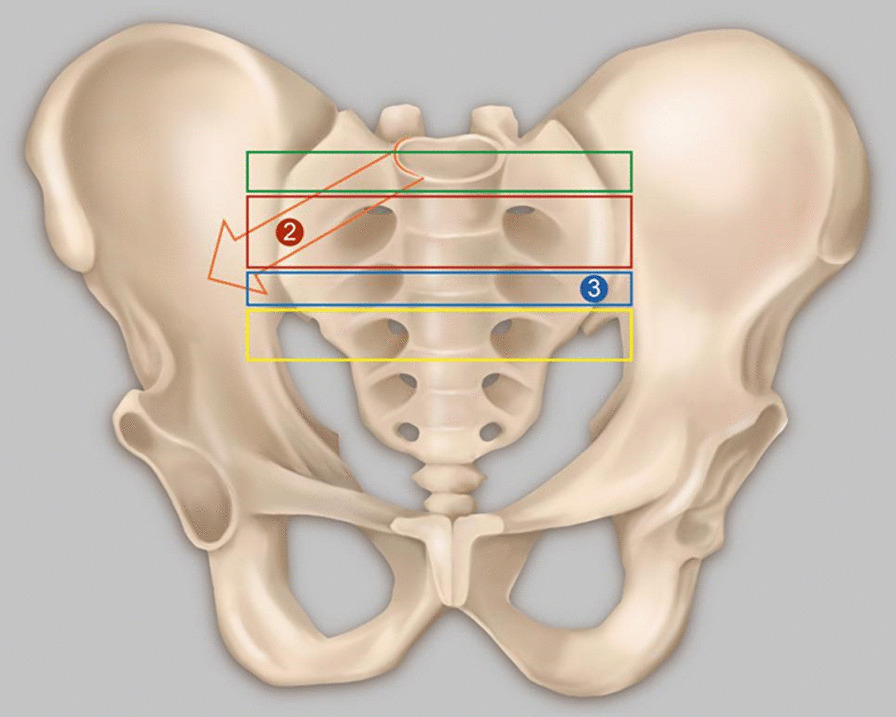
Definition and zoning of the distal and proximal sacroiliac joints. Each level of CT plain scan from the sacroiliac joint emergence to the sacral 3 foramina through five markers under the boundary was divided into four zones: sacroiliac joint emergence, the widest spacing between the sacral slope and the sacral nerve trunk canal, the disappearance of the lower boundary of the sacral 1 foramen, the disappearance of the lower boundary of the sacral 2 foramen, and the disappearance of the lower boundary of the sacral 3 foramen. The level between the beginning level of sacroiliac joint emergence and the maximum spacing between the sacral slope and the sacral nerve root canal was zone 1, etc., divided into four zones

Norio Yamamoto et al. [19] detected VP at the pubic and sacral fractures, but not at the iliac or ischium fractures. Hematoma at the fracture site located in a closed space creates a negative pressure environment for VP generation [19]. Our study revealed that most VPs are located in the sacroiliac joint, almost all in the sacroiliac joint area near the sacral foramen (Fig. 1). One case is located at the pubic symphysis (Fig. 2), and another at the medial side of the iliac bone (Fig. 3), suggesting that VP formation is often based on a complete joint or relatively closed space. We will further analyze the VPs that appear in the sacroiliac joint and select the upper, middle, and lower points of the sacroiliac joint for zoning and observation. Most VPs are located in the front 1\/3 area, some continue to the middle 1\/3 area, and almost none are located in the rear 1\/3 area (Fig. 4). Therefore, we suspect that it may be related to the anatomical mechanics, wherein the front is the compressive stress side of the pelvis and the rear is the tensile side. Degeneration instability is considered caused by long-term compressive stress or buffering protection. Then far and near ends of the sacroiliac joint were defined and divided: on all levels of CT plain scan. It is divided into 4 areas through the lower boundary of 5 marks from the appearance of the sacroiliac joint to the sacroiliac 3 hole: the sacroiliac joint appears, the sacrum slope and the sacral nerve trunk are the widest, the lower boundary of the sacroiliac 1, 2, and 3 holes disappears. From the initial level of the sacroiliac joint to the level between the sacral clivus and the maximum distance between the sacral nerve root canal is zone 1, etc., which is divided into four zones (Fig. 5). Most VPs were located in zone 2, and a small number of VPs were located or involved in zones 1 and 3, and zone 4 was rare. Almost all of them are in the sacroiliac joint area near the sacrum foramen, especially in the sacroiliac joint area near the sacrum 1 foramen and the compact area in front of the sacroiliac joint. This area is preliminarily considered to belong to the mechanical transmission area, and the sacroiliac joint transmits the axial force generated by the spine to the lower limbs. The surface of the sacroiliac joint is flat, and the direction is almost parallel to the maximum load plane [20]. It may cause more degenerative changes than other load-bearing joints, and is the most prone area to degeneration. The sacroiliac joint area of the sacroiliac 1 screw channel was almost not observed near the slope.

A study reporting delayed or nonunion limb fractures considered VP detection at the fracture site as a marker of delayed or nonunion [6]. VP only existed at the fracture site in patients with limb nonunion, with no gas in the adjacent soft tissue. Our study surgically treated 227 patients in the high-energy trauma group, and the healing time of 201 bone union fractures was 9.8 ± 5.3 weeks. Additionally, 54 patients were surgically treated in the FFP group, and the healing time of 49 bone union fractures was 9.3 ± 3.8 weeks.

This study has some limitations. First, the sample size of VP in pelvic fractures is relatively small, which is not enough to detect the factor differences between groups. Secondly, clinical management lacks consistency in this retrospective single-center study. Third, the data were missing, and some patients were lost to follow-up. Finally, this study only explored the phenomenon of VP in patients with pelvic fractures, but VP can occur in healthy groups. Therefore, a second control group consisting of healthy individuals was lacking in the control group design. Multi-center, prospective, and large sample clinical research remains necessary in future for the incidence and clinical significance of VP in closed fracture, which is of great benefit to deepen the understanding of VP and guide clinical work.

The incidence of VP in pelvic fractures is statistically high, but VP in pelvic fractures may be influenced by a variety of factors such as examination technique, joint location, and population composition. Therefore, VP is not a reliable indication of pelvic injury. Clinically, we need to determine the nature of VP in conjunction with gas patterns, laboratory tests, history, and physical examination. A better understanding of this anatomical phenomenon can prevent misdiagnosis and prevent patients from receiving suboptimal treatment.

## Data Availability

The datasets are available from the corresponding authors on reasonable request.
